# Physiotherapists’ views of implementing a stratified treatment approach for patients with low back pain in Germany: a qualitative study

**DOI:** 10.1186/s12913-018-2991-3

**Published:** 2018-03-27

**Authors:** Sven Karstens, Pauline Kuithan, Stefanie Joos, Jonathan C. Hill, Michel Wensing, Jost Steinhäuser, Katja Krug, Joachim Szecsenyi

**Affiliations:** 1 Department of Computer Science; Therapeutic Sciences, Trier University of applied Science, Trier, Germany; 2 Department of General Practice and Health Services Research, University Hospital Heidelberg, Heidelberg, Germany; 3 Department of Therapeutic Sciences, SRH Hochschule Heidelberg, Heidelberg, Germany; M.Sc. Sport Physiotherapy, German Sport University Cologne, Cologne, Germany; 4 Institute of General Practice and Interprofessional Care, University Hospital of Tuebingen, Tuebingen, Germany; 5 Research Institute of Primary Care and Health Sciences, Keele University, Keele/Stoke-on-Trent, UK; 6 Institute of Family medicine, University Hospital Schleswig-Holstein Campus Luebeck, Luebeck, Germany

**Keywords:** Stratified care, Physical therapy, Low back pain, Health services research, Qualitative research

## Abstract

**Background:**

The STarT-Back-Approach (STarT: **S**ubgroups for **Tar**geted **T**reatment) was developed in the UK and has demonstrated clinical and cost effectiveness. Based on the results of a brief questionnaire, patients with low back pain are stratified into three treatment groups. Since the organisation of physiotherapy differs between Germany and the UK, the aim of this study is to explore German physiotherapists’ views and perceptions about implementing the STarT-Back-Approach.

**Methods:**

Three two-hour think-tank workshops with physiotherapists were conducted. Focus groups, using a semi-structured interview guideline, followed a presentation of the STarT-Back-Approach, with discussions audio recorded, transcribed and qualitatively analysed using content analysis.

**Results:**

Nineteen physiotherapists participated (15 female, mean age 41.2 (SD 8.6) years). Three main themes emerged, each with multiple subthemes: 1) the intervention (15 subthemes), 2) the healthcare context (26 subthemes) and 3) individual characteristics (8 subthemes). Therapists’ perceptions of the extent to which the STarT-Back intervention would require changes to their normal clinical practice varied considerably. They felt that within their current healthcare context, there were significant financial disincentives that would discourage German physiotherapists from providing the STarT-Back treatment pathways, such as the early discharge of low-risk patients with supported self-management materials. They also discussed the need for appropriate standardised graduate and post-graduate skills training for German physiotherapists to treat high-risk patients with a combined physical and psychological approach (e.g., communication skills).

**Conclusions:**

Whilst many German physiotherapists are positive about the STarT-Back-Approach, there are a number of substantial barriers to implementing the matched treatment pathways in Germany. These include financial disincentives within the healthcare system to early discharge of low-risk patients. Therapists also highlighted the need for solutions in respect of scalable physiotherapy training to gain skills in combined physical and psychological approaches.

**Electronic supplementary material:**

The online version of this article (10.1186/s12913-018-2991-3) contains supplementary material, which is available to authorized users.

## Background

In Western Europe and worldwide, low back pain (LBP) is a leading cause for years lived with disability [1, 2]. This has enormous economic consequences for healthcare expenditures and loss of work productivity [3, 4]. The German physiotherapy caseload consists predominantly of patients with LBP [5, 6], in which a key challenge is to identify patients at risk of an unfavourable prognosis [7]. Early assessment and treatment of those at high risk is not only important in terms of improving clinical outcomes and cost effectiveness, but also in reducing the impact of LBP, including individuals’ health, physical and social activities and local and global participation [8, 9].

Research has demonstrated that standardized risk-specific stratified treatment approaches could be superior to typical physiotherapy practice [10, 11]. Several instruments have been developed to assist in risk screening, including the STarT-Back-Tool (SBT) and the Orebro Musculoskeletal Pain Screening Questionnaire, which are both internationally recognised and can provide accurate prediction of LBP disability. The SBT was specifically developed for use in primary care, and the Orebro has strengths for use in an occupational health setting [12–14].

The STarT-Back-Approach (**S**ubgroups for **Tar**geted **T**reatment) uses the validated SBT to provide risk stratification for primary care patients with LBP into three risk subgroups (low risk, medium risk, high risk) with respect to likelihood of an unfavourable therapy outcome [10]. The tool is available in more than thirty languages and consists of nine items, with the first four relating to biomedical factors and the last five identifying modifiable psychosocial risk factors [14, 15]. In 2012, it was translated and cross-culturally adapted into German, following internationally accepted guidelines [guidelines available from: [16–19]. In the STarT-Back-Approach, patients are first stratified using the SBT and then provided with recommended treatments that match each subgroup’s risk profile.

The recommended treatments for patients at low risk on the SBT involve the provision of reassurance, advice and self-management support, with attention paid to ensure that patients are not over-treated or over-investigated. Patients at medium risk, in addition to the minimal package, receive evidence-based physiotherapy treatment incorporating exercise and manual therapy. Patients at high risk receive psychologically informed physiotherapy, which integrates physical and psychological treatment approaches with the aim of reducing obstacles to recovery such as unhelpful beliefs and illness behaviours. In the UK STarT-Back clinical trial, the physiotherapists who provided treatment for high-risk patients received training over six days as well as ongoing monthly mentorship from a physiotherapist specialised in chronic pain and a clinical psychologist with pain management expertise [10, 20]. The views of physiotherapists who received this training were reported and highlighted their appreciation of their need for new skills to better address psychosocial barriers to recovery and to increase their confidence in managing patients with complex psychosocial needs [21].

A randomized controlled trial provided evidence that the STarT-Back-Approach to managing LBP in primary care provided significant benefits such as cost effectiveness and reductions in pain and disability in comparison to typical physiotherapy [10]. Following the dissemination of this research, there has been widespread adoption of the approach among spinal pathways and physiotherapy practices in the UK [22]. However, despite evidence for the benefits of this approach, implementation of the STarT-Back-Approach in Germany has not yet occurred. Implementing this approach in Germany may provide an opportunity to improve the treatment of patients with LBP and help address deficits in current LBP services, such as the scarcity of multimodal pain programs in many areas [6, 23, 24]. However, it may also not be possible due to differences between the healthcare systems in the UK and Germany.

One concern about adopting the STarT-Back-Approach in Germany relates to differences in the structure and organisation of physiotherapy [25–27]. For example, physiotherapists in the UK require an academic degree, whereas German physiotherapists are generally trained at the vocational level, with only a minority being trained at universities [28]. The proportional number of physiotherapists is nearly three times as large in Germany, which has 183,000 therapists for 83 million people (or one therapist for every 454 people), whilst the figure in the UK is 52,426 therapists for 66 million residents (or one therapist for every 1259 people) [29, 30]. Other differences include the free UK National Health Service (NHS), which pays physiotherapists a fixed salary to treat LBP patients in their locality, whilst approximately 90% of patients in Germany have statutory health insurance [31], which funds LBP treatment according to the number of physiotherapy visits made. In addition, to treat a person with LBP, German physiotherapists need a referral from a physician, whilst in the UK physiotherapists are recognised as qualified first-contact practitioners ([32], for a detailed comparison Germany/UK on referral see [33], pages 5 and 6). In Germany, the LBP referral process to physiotherapy is defined by a directive which includes a detailed referral catalogue. Referrals for physiotherapy are most often written by general practitioners (GPs), but a substantial number also come from orthopaedic specialists [34]. To regulate physician referral behaviour, there are financial penalties assessed to those who refer excessively. This is often correlated with clinician dissatisfaction [35]. To make valid LBP referrals for physiotherapy, physicians must clearly define the treatment aims, frequency (sessions per week) and prescribed approach (e.g., manual therapy, exercise combined with electro- or thermotherapy). A standard LBP referral from a GP in Germany typically asks for up to six treatment sessions of fifteen to twenty-five minutes each. When patients attend physiotherapy clinics, their appointment scheduling is supposed to follow the physician’s stipulations on the referral form with any further treatment sessions (up to eighteen) requiring additional physician approval [6].

Given these differences in the German healthcare system for patients with LBP, we previously reported the views and perceptions of German primary care physicians about implementing the STarT-Back-Approach [34]. This work identified that, whilst participating physicians overall were positive about the STarT-Back-Approach, they felt that considerable care and attention would be required to successfully implement the approach. The need for improved inter-professional collaboration between physicians and physiotherapists and uncertainty about the knowledge and skills of German physiotherapists in managing complex patient needs were specifically highlighted concerns [35].

Following a phenomenological approach, we aimed to explore with German physiotherapists their perspectives on implementing the STarT-Back-Approach. We worked to understand the perceived potential organisational barriers to and enablers of using this approach within their practice.

## Methods

### Design

Three two-hour workshops were conducted at a university hospital. The STarT-Back-Approach was presented by a member of the study team, followed by a focus group discussion [10, 36]. This qualitative methodology (focus groups) was chosen since it enables participants to express themselves openly, with the added value of social interaction stimulating conversation and the development of ideas [37].

The workshops were led by a trained and experienced facilitator (SK, researcher, educator with experience in graduate and post-graduate pain-management training) and a co-facilitator (PK, a musculoskeletal physiotherapy clinical lecturer). The workshops began with a presentation about the STarT-Back-Approach and related research with input from the original UK developers [10, 14] (see Table 1 for a description of the presentation content). The focus group discussion that followed used a semi-standardized interview guideline to structure the conversation (see Additional file 1), during which the co-facilitator took detailed notes. All participants gave written informed consent before participation, and ethical approval was obtained from the Ethics Committee of the University of Heidelberg (Registration ID: S-414/2013).

**Table 1:** Content of the workshop’s introductory presentation

Risk factors for LBP	Categories
	Impact
STarT-Back-Tool	Development and content
	Scoring the tool
STarT-Back-Approach	Treatment pathways for patients at low, medium and high-risk
	Results of the STarT-Back-trial, improvements in disability at 6 and 12 months
German GPs’ views of the STarT-Back-Approach	Key findings from similar focus-groups with GPs [35]

*GP* General Practitioner

### Participants

The participating physiotherapists were recruited via three different networks: a regional group of the German physiotherapy professional association, participants in a local journal club and members of a regional educational network. After initial contact via email or telephone, invitation letters were sent with information about the STarT-Back-Approach attached [10, 38]. Participation was voluntary, and no financial incentives were provided. Attendees were arranged heterogeneously in terms of academic and post-graduate training, working experience and main field of practice in order to build a balanced platform for the discussions [39].

### Data analysis

The discussions were audio recorded and completely transcribed. Transcription was carried out (PK) verbatim following predefined standards [40], with transcripts checked after completion in comparison to the audio files by two of the authors (SK, PK).

The transcript coding was structured using a content analysis approach. With reference to Krippendorff (1969), Mayring defines “content analysis as the use of replicable and valid method for making specific inferences from text to other states or properties of its source” [41, 42]. Initially, a deductive category system with detailed definitions for each category was produced in alignment with the interview guideline and reflecting the major concepts for implementation: the intervention, the inner and outer setting and the individuals involved. The fifth concept of “implementation process” was not a separate focus of the discussions or data analysis but is featured in the discussion. One of the three workshops was chosen at random with the transcript independently coded (by PK and SK) and compared; any resulting discrepancies were discussed and clarified. Coded categories were repeatedly and iteratively adapted for all three focus groups and checked for relevance against the Consolidated Framework for Implementation Research (CFIR) [43] due to its relevance to the project research aims [43–46]. Nevertheless, a slight adaptation of the CFIR framework was made [43] to improve coherence, with the “inner setting” and “outer setting” subthemes combined under one main theme of “setting.” To assist the coding process, we used the R software with the RQDA package (http://rqda.r-forge.r-project.org/; [47]).

## Results

A total of 19 physiotherapists (median age category of 40 to < 50 years, 9 younger, 5 older) participated in the three focus groups, with the following gender female/male ratios, respectively (8/0, 5/2 and 2/2). All participants were experienced in treating LBP, with length of experience ranging from four to 29 years. A third (32%) of participants had academic-level qualifications, while two-thirds (68%) had a post-graduate certificate in manual therapy. Participants worked in a range of settings, most within the public health care system: physiotherapy clinics (*n* = 15), hospitals (*n* = 3), rehabilitation centres (*n* = 2), as educators (*n* = 4), as a researcher (*n* = 1) or as freelance or self-employed clinicians (*n* = 3).

A number of subthemes emerged and were categorised under the following three main theme headings: 1) the intervention characteristics (14 subthemes), 2) the healthcare setting (24 subthemes) and 3) individual patient characteristics (9 subthemes); they are listed in Tables 2, 3 and 4. Quotations underpinning the meaning and variety of the subthemes are provided in Additional file 2. The following paragraphs summarise the key issues that emerged from the discussions.

**Table 2:** Intervention Characteristics: CFIR-Topics and developed subthemes

Topic^a^	Subthemes
Evidence strength and quality	− Treatment duration
Relative advantage	− Matching patients − Benefits of classification − Advantages of SBT − Objectiveness
Adaptability	− Implementation of classification − Time point of SBT application
Complexity	− Chances of Implementation − Implementation of classification − Consumption of time − Intensity assessment and advice
Design quality and packaging	− Structure SBT − False Classification − Treatment description − Standardisation

^a^based on the Consolidated Framework for Implementation Research (CFIR) [43]

*SBT* STarT-Back Tool

**Table 3:** Setting: CFIR-Topics and developed subthemes

Topic^a^	Subthemes
Structural characteristics	− Chances of implementation − Manual therapy − Requirements for staff
Culture	− Profitability inner setting − Manual therapy
Implementation climate	− Chances of implementation − Profitability inner setting − Current system − Role of PT − Allocation of competencies
Readiness for implementation	− Organisation in clinic
Design quality and packaging	− Profitability inner setting
Patients’ Needs and Resources	− Professional policy − Patients‘self-commitment − Patients‘views on treatment scope − Patients‘rethinking − Patients‘expectation on passive treatment − Patients‘reaction to classification − Misinformation − Patients‘status
Cosmopolitanism	− Interprofessional collaboration − Collaboration with GP
Peer pressure	− Implementation of interprofessional collaboration − First contact − Competencies perceived by others − Role of PT within the healthcare system − Patients’ reaction to classification − Allocation of competencies
External policies and incentives	− Remuneration − Profitability outer setting − Professional policy − First contact − Role of PT within the healthcare system

^a^based on the Consolidated Framework for Implementation Research (CFIR) [43]

**Table 4:** Individual characteristics: CFIR-Topics and developed subthemes

Topic^a^	Subthemes
Self-efficacy	− Role of Physiotherapist − Competencies in management of complex patients − Self-efficacy − Training psychosocial aspects
Individual identification with organization	− Commitment
Other personal attributes	− Competencies in management of complex patients − Work experience − Addressing psychosocial aspects − Commitment − Academic qualification − Acceptance by novices − Training of psychosocial aspects

^a^based on the Consolidated Framework for Implementation Research (CFIR) [43]

### Intervention characteristics

In general, participants endorsed the concept of stratified care and its implementation in Germany. Participants’ perspectives on treating patients with the STarT-Back-Approach varied. Some stated that the approach would be “daily business”, whereas others stated that a substantial adjustment of their usual LBP treatment would be required.

PT7–1 *“I think it’s a good approach and a necessary approach.”*

PT2–2 *“I think [treatment as described for the STarT-Back-Approach] that’s what we’re doing day after day anyway.”*

PT3–2 *“That’s an important approach that currently probably doesn’t happen in many practices or treatment centres.”*

The benefit of subgrouping patients was widely accepted as a means of increasing clinical effectiveness and for highlighting the relevance of psychosocial barriers to recovery to physiotherapy practice. The strengths and limitations of clinicians’ using their intuition alone to provide individually tailored treatment in contrast to the use of the SBT-recommended treatments for low-, medium- and high-risk treatment groups were discussed. However, several physiotherapists viewed the “classification” of patients into three subgroups negatively due to a perception that this would reduce their focus on each patient’s individual needs.

PT1–3 *“I find it hard to pigeonhole patients like that. […] but I don’t see a problem from a therapeutic point of view, […] because physiotherapy up to now has been quite intuitive.”*

The length of the SBT was discussed, with concerns expressed that it was not comprehensive enough, with some items missing (e.g., pain severity), whilst others felt its brevity made it more feasible and easy to use. Another topic was the STarT-Back-Approach’s emphasis on addressing psychosocial obstacles leading to more successful clinical outcomes. Participants agreed that educating patients and supporting their self-management and self-reflection would be more effective, provided that their LBP was appropriately monitored. Some therapists suggested there should be a mechanism for providing further assistance to low-risk patients who fail to respond to the minimal intervention package.

PT3–3 *“[…] it means you’ve got a totally different starting point with the patient, […] the patient is proactive and we can get away from never-ending treatment sessions.”*

PT4–2 *“But I think every one of us knows this sort of nice patient who can still function but still suffers from something.”*

Concerning the practicalities of including the SBT in daily clinical practice, some participants suggested that patients should complete it at home or in the waiting area prior to their appointment with help of an assistant. Others suggested it should be used more interactively during the consultation as part of the clinical assessment.

PT4–2 “*Ideally in future the patient should bring the completed questionnaire with them. I would like it like that. […]”.*

Discussions about the recommended targeted treatments were equivocal. Some participants asked for further treatment specification and standardisation. For example, both the definition and content related to manual therapy and neurological examination were discussed and found to be interpreted very differently. Others, meanwhile, welcomed the broad nature of the approach, as it provides ample individual freedom but remains evidence-based.

*PT6–1 “It’s positive in my opinion too, that there is a systematic approach, having a kind of guideline. A starting point.”*

The low number of treatment sessions, which is below the current average, and the subsequent treatment success achieved by participants within the STarT-Back-trial, surprised some of the participants.

*PT3–3 “[...] if I would have such an improvement after four treatment sessions, not needing eighteen and having no difference [in treatment outcome].”*

### Setting

The number of treatment sessions provided per patient was discussed with some animation because of the potential monetary consequences. Some feared a reduction in the number of treatment sessions that might be stipulated for patients, while others anticipated better compensation due to improved efficiency and a concomitant increase in patient satisfaction. Long-term patients were discussed as financially rewarding, as they are a consistent source of income for some clinics. However, some participants advocated that good-quality treatment and more rapid improvement for patients should take priority. Others expressed the fear that the demand for physiotherapists to manage LBP could decline or even be eliminated because of psychosocial obstacles to recovery that were outside their remit.

PT2–2 *“I think if word gets around that it’s not just about getting as many referrals as possible and treating the same thing every week for years [that is positive]. Instead you say ‘this is what it’s about, now off you go to the sports club’ or something like that.”*

PT3–3 *“[…] the more you as a physiotherapist address these psycho-social aspects of medicine, don’t you risk rationalising yourself out of the picture?”*

Another monetary aspect discussed was the potential implications on physiotherapists’ wages of implementing the approach. A potential rise in wages, justified by the profession’s taking on new and greater responsibilities and skills and working with greater efficiency, was seen as a possible benefit from an implementation. A comparison was made with higher reimbursements recently established as part of a particular health insurance pain prevention programme. Participants complained about the current treatment compensation system used by German health insurance companies, stating that they preferred a lump-sum payment to being remunerated according to the details of each physician’s referral.

*PT1–3 “I think reimbursement is rather cause of frustration for all of us therapists [laughter], we are convinced that we don't get paid to an extent we think we are qualified [...]. With these tasks [described for the STarT-Approach], with these additional qualifications, physiotherapy is gaining more, dramatically more importance and thus deserves a higher reimbursement.”*

From a professional policy point of view, participants showed high levels of frustration with the current system and would welcome any improvement of working conditions. Moreover, the participants demanded an improved professional status for physiotherapy within the healthcare system and more robust lobbying efforts on their behalf. The STarT-Back-Approach was seen as a possible flagship that might lead to increased responsibilities, improved shared decision-making and freedom to choose treatment modalities.

PT3–3 “*That’s why I think it’s a good idea to think through the whole concept that we have in Germany, and re-examine it. [...] How it’s carried out, the restrictions for doctors and suchlike.”*

*PT1–3* “*[…] that would indeed be a first flagship. Meaning, that in medicine or even politics they would say: ‘look how important physiotherapy is!’”*

It was suggested that German health insurance companies should adjust their payment mechanisms for physiotherapists in order to give them greater freedom in decision-making, especially in relation to treatment time, duration and frequency.

*PT2–1 “I think that’s a thrilling topic, and also necessary, but from the view of our own professional identity we really do something completely different.”*

The networking aspects within the STarT-Approach were seen as necessary and helpful for improving inter-professional collaboration and simplifying the referral process. It was stated that establishing greater inter-professional collaboration might be easier with this approach.

PT3–1 *“And he [physician] gives it the nod because he knows what I’ll be doing next, like that. I don’t think you should scatter this about so that everybody gets involved; better to work with an established partner.”*

In addition, the potential of a shared treatment approach based on knowledge of an individual’s risk status was considered a possible way of facilitating inter-professional collaboration. However, whilst it was noted that relationships between physiotherapists and GPs might be improved, in this context, participants hypothesised that GPs’ limited levels of trust in physiotherapists’ work and education might be a significant challenge.

PT7–1 *“[…] and our everyday reality is that there is little confidence in our capabilities […] and in my opinion, this approach enhances our own confidence as well as the physicians’ trust in us, that the patients receive appropriate physiotherapy treatment.”*

Patient expectations were discussed as an important influencing factor. Some participants felt that patients would be astonished by the change in treatment approach. The view was shared that some patients favour passive treatments and lack in intrinsic motivation, not really wanting to return to work as fast as they could.

*PT2–2 “[…] no one [patients] is used to it so far. It could be met with amazement to start with.”*

*PT7–2 [roleplaying] “She didn’t even let me get undressed [laughs]. She just asked me things.”*

*PT5–1* “*I mean it depends on the patient. If he is really motivated and wants to recover quickly, then things will go faster […], for those people who enjoy being given a sick note and spending a week or two at home […]. You have to call it what it is, there are people who like that.”*

Another potential obstacle was difficulty arising from contradictory patient information given by clinicians with a strongly bio-structural diagnostic approach. Equivalent misleading information spread via mass media were also discussed. Moreover, the possibility of an emerging conflict between patients who already had established chronic pain behaviours, the reluctance of physiotherapists to treat them and therapists being uncertain about when a referral for further psychological treatment might be indicated were all considered. This last point reflecting the reasonableness of indefinite bio-medically justified treatment.

*PT2–2 “Basically we are getting to a certain point when we have to realise that [the patients] shouldn’t see a physio anymore, rather sending them to a back pain prevention programme or a back exercise group.”*

Another issue was whether a homogenous team of therapists in each clinic would be necessary to deliver a STarT-Back-Approach, or whether an alternative model with patient risk status matched to junior or senior therapist status would be better. Moreover, participants felt that being able to provide all three treatment pathways might be challenging for smaller clinics. They encouraged the idea of developing health care centres.

PT3–3 *“I think that in England you’ve got this thing with Juniors and Seniors […]. As a Junior you can look after the low-risk cases and then later on becoming a Senior you can work with the high-risk cases. Perhaps a structure like that would be desirable.”*

PT1–3 *“Even not every physio has to be a specialist of some sort, I think that’s clear. Perhaps it would be sufficient when in your centre there would be one or two experts who do the first session and then, depending on the risk status, pass the patient on to a junior therapist according to the therapeutic approach, recurrence rate.”*

Other aspects concerned the clinic structure and the process of appointment scheduling. The need for rooms with solid walls, instead of cabins with curtains, to offer privacy for psychosocial treatments was discussed, as was the idea of time intervals specifically blocked for patients with LBP could be an opportunity to avoid long delays for initial appointments.

PT3–3 *“Who has got these sort of facilities [needed for the STarT-Approach]? Who’s got a room with a door? And not a room with three benches and a curtain [in between], for example.”*

PT4–2 *“[…] I could imagine setting aside blocks of 2 or 3 hours doing one of these after another […].”*

### Characteristics of individuals

Current levels of physiotherapy qualification and training required were the main points discussed. Participants liked the increased responsibility in patient guidance.

*PT3–3 “So I could see myself in the role of somebody pointing the way forward, a sort of coordinator like nurses in the hospital.”*

Appropriate communication and education skills were reported by some therapists to already be in place. Others asked for additional support, including input from an interdisciplinary team for high-risk patients. They described the current focus on hands-on treatment techniques and therefore a lack of appropriate skills and previous training in dealing with psychosocial obstacles.

*PT6–2 “[...] and I think we are actually already trained. To take into account this aspect with the high-risk this psychosocial aspect. I see this again and again that it’s more useful to spend 20 minutes talking and not doing manual therapy.”*

Work experience was discussed between the alternate views around compensating for a lack of psychosocial specific training and this reality in combination with reduced reflective skills being a disadvantage. Others highlighted the importance of therapists’ clinical experience in being able to address psychosocial aspects.

*PT4–3 “I think, yes, then it is exactly right to work on psychosocial aspects, too. That's what you only learn working with the patient, you only learn by experience.”*

Participants felt that in order to implement the approach, there was a need for an appropriate high-risk training programme covering all the key aspects of psychologically informed practice.

*PT3–2 “But it’s definitely true that it’s something that not everybody can do [skilled communication] and it’s hard to learn it on a theoretical basis. I think you’ll need supervision on top, [...]”*

The qualifications required of physiotherapists to treat patients using the STarT-Back-Approach was repeatedly verbalised as something that was beyond the average level currently available among German physiotherapists. Academic programmes were seen as having the advantage of being trusted to fully cover the relevant topics required.

*PT4–3 “When we are talking about vocational training. And I’ve just visited two [schools], it’s all technique, technique, technique. Now there are academic programmes in addition, 40 or 50 of them [at universities], which have more of that stuff included: clinical reasoning and all this stuff. There are vocational schools which still don’t teach evidence-based working. Yes, I guess in this respect an academic graduate has an advantage.”*

*PT1–3 “The basic assessment [described for the STarT-Back-Approach], well, reflecting my vocational training, I wouldn't have seen myself qualified to do this. That only came later. [I don't know what it's like nowadays] I hope it improved, specifically neurological examination, to differentiate and so forth.”*

Participants felt that one indication of suitable therapists who were likely to be motivated to treat high-risk patients was to begin by identifying those that had completed several post-graduate training courses.

*PT1–3 “I think that’s right as well. Too few physiotherapists who are reasonably committed. When I say committed, I mean the ones who regularly upgrade their skills and aim at offering better therapy.”*

## Discussion

The aim of the study was to explore the views and perceptions of physiotherapists regarding the implementation of a stratified care approach to managing LBP in primary care in Germany. The STarT-Back-Approach differs from current daily practice in the use of a questionnaire to stratify patients and in varying the intensity of physiotherapist-patient relations; it even alters the very scope of physiotherapy practice. Nevertheless, there are physiotherapists would appreciate its implementation and who express self-confidence in engaging with the approach, providing the framework is adapted to make it appropriate for the German healthcare system.

The SBT was accepted as a useful guide by several participants as a way of supporting their clinical reasoning, rather than as a strict protocol for treatment. However, as part of its implementation, due consideration of adaptations required to fit the broader healthcare context might also be necessary so that clinics would not be financially disadvantaged from discharging low-risk patients early and, conversely, so that sufficient treatment session times would be available for high-risk patients. Additional points identified as important challenges for implementation included the heterogeneous qualifications of German physiotherapists and the perception that a stratified approach could mean sacrificing intuitive treatment. The adaptation of current usual physiotherapy for patients at medium risk was expected to be fairly straightforward.

Stratification of patients using the SBT is a major component of the STarT-Back-Approach, but the utilisation of questionnaires in clinical practice is not currently common practice in Germany. Some participants were reluctant to use the approach, as they felt it could pigeonholing the patient. Others argued that a differentiation in treatment approach for patients with a shorter or longer history of complaints might be necessary. Like the discussions with physicians [35, 48], controversial aspects included the time required for SBT administration and its potential influence on professional-patient communication. Some recommended that patients be stratified before the consultation, while others indicated their preference for using it as part of their clinical assessment.

Reflecting on the aspects of working experience and pigeonholing, it can be argued that the STarT-Back-Approach allows for the therapist to overrule the assignment of a patient to a given risk level. Even though some of our participants feared misclassification, Hill et al. reported that physiotherapists rarely made use of the possibility of overruling the treatment allocation resulting from the questionnaire [10]. In the literature, the influence of psychosocial factors on chronification and response to treatment is described comprehensively, and physiotherapists at least partially recognise it [49–52]. The participating physiotherapists agreed with the consideration of psychosocial factors as a dominant obstacle in the rcovery of patients with LBP and liked this aspect of the STarT-Back-Approach. They also saw the opportunity for fast-tracking patients to physiotherapy as very positive and thus a facilitator for implementation.

Treatment within the STarT-Back-Approach is in line with the basic principles of evidence-based practice, incorporating the latest research knowledge but also giving therapists the ability to consider their own therapeutic strengths and the preferences of their patients. Nevertheless, the workshop participants argued on the issue of individualisation and standardisation, asking for more detailed specifications about the content of the STarT-Back-Approach and discussing the benefits of the freedom to choose different treatment contents within the boundaries set for patients at low, medium and high risk of persistent disabling LBP. Manual therapy techniques were a prominent subject in this particular discussion, possibly reflecting the post-graduate training of the participants.

Within the STarT-Back-Approach, a one-off clinical appointment is described for low-risk patients [10], but research indicates that GPs seeing patients first in the German primary care system will not have the time resources to conduct this task comprehensively [27, 35]. This means that improved inter-professional collaboration is needed in order to deliver a high-quality minimal treatment package. Like the physicians interviewed previously, the physiotherapists pointed out the need for specifically coordinated appointments for low-risk advice sessions, if implemented in physiotherapy clinics, to guarantee quick service [35]. The advantages of medical centres with co-located physiotherapy and physician practices were part of the discussion. This would mimic UK NHS centres to some extent, and the relevance of this aspect might be explored in future studies.

Participants also highlighted adaptations of a number of different external influences on healthcare. German physiotherapy clinics are usually privately owned. They depend on referrals from physicians who most often prescribe therapy in sets of six sessions, with each session being remunerated equally. Reflecting these structures, the aim of the STarT-Back-Approach to protect patients against over-treatment would result in a substantial reduction in earnings. Participants noted that the current German healthcare system offers no direct benefit for clinics to discharge patients early. Therefore, implementation of incentives that reward early discharge of patients would be needed to facilitate implementation of this approach.

Another practical issue reported by German physiotherapists as a negative influence on the quality of treatment is the allotted treatment time [53]. A basic remuneration position is defined with a treatment time of 15 to 20 min per session. The 45-min duration of the UK high-risk treatment-session in the STarT-Back trial exceeds this standard by more than a factor of two. One solution built on current structures might be to allow double appointments for high-risk patients, although under current regulations, this strategy would be difficult to implement, since statutory health insurance does not currently allow double appointments. A current remuneration position with a defined treatment time of 60 min is given for patients with “complex injuries/impairments” (“D1”). However, remuneration of around 35 Euro would not be sufficient for clinics to provide complex bio-psychosocially orientated treatment.

The orientation of the physiotherapist as biomedical or bio-psychosocial influences the treatment approach chosen [54]. Within the current definition of physiotherapy as described by the World Confederation for Physical Therapy (WCPT), physiotherapists “help people maximise their quality of life, looking at physical, psychological, emotional and social wellbeing” [55]. Still, the relevance of cognitive, psychological and social factors is not fully recognised by therapists [52]. This was reflected by the participants, who said that many physiotherapists might have to rethink their concept of physiotherapy, since the biomedical paradigm plays a dominant role in current Germany physiotherapy practice. Nevertheless, like UK physiotherapists, the participants acknowledged psychosocial factors as obstacles to recovery [21]. This indicates a potential enabler for the implementation of a corresponding approach. On the other hand, and also like UK therapists, participants feared that some of their LBP patients might not be willing to accept a therapy incorporating a comprehensive amount of education and advice, because of the aforementioned dominance of biomedical approaches [44]. This perspective is supported by descriptions of the impact of patient preferences on treatment and research reporting that stronger patient involvement in bio-psychosocial approaches is perceived as challenging [54, 56]. Patients were described as still having outdated expectations of physiotherapy and health care more generally, where the patient is passive and treated by the medical professional to fix a biomedical problem [57, 58].

In line with the preference of many physiotherapists to deal with biomedical factors, some participants were sceptical about the ability of German physiotherapists to treat high-risk patients [52], stating their preference for a collaborative approach with psychologists. In reflecting on this perception, it has to be noted that these therapists have not had any specific training in treating high-risk patients and that access to psychotherapy was described as severely limited in the current system [35]. In addition, previous research suggests that high-risk treatment training can encourage physiotherapists and improve their confidence in treating these patients [44, 59, 60]. Sanders et al. found that trained physiotherapists described a widened horizon, that they were successfully using their newly learned communication techniques and that they had improved patient interactions [44]. Moreover, they described an increased self-confidence and self-reflection, leading to improved clinical decision-making with patients in complex situations. Synnott et al. correspondingly demand easily accessible training [60]. Following the procedure developed for the STarT-Back clinical trial, this would require six days of training including formal trainer-led teaching, experiential learning, role playing and mentoring sessions [61]. In this sense, implementation of the STarT-Back-Approach and corresponding training of therapists could become an important vehicle for change in Germany’s physiotherapy profession.

Designing the implementation process corresponding to the category identically named by Damschroder et al. [43] was not the emphasis of the focus groups conducted. Nevertheless, some approaches described in the literature can be compared with the material reported in this study. For example, different facilitators for implementation of the STarT-Back-Approach were identified that are common to other implementation initiatives: training programmes, adaption of workload, interventions using opinion leaders or knowledge brokers, conducting workshops, encouraging journal clubs and giving presentations [62]. In line with this, the most prominently discussed facilitator was the participants’ perceptions of the need for appropriate training programmes including knowledge about psychosocial predictors, methods of communication to elicit and address unhelpful LBP beliefs and illness behaviours and appropriate ongoing mentoring. In general, participants agreed that communication training does not receive adequate recognition by German physiotherapists. Here, involvement of knowledge brokers and opinion leaders could be very beneficial [62].

A topic of discussion not specifically fostered by the guideline developed for the interviews, but which repeatedly emerged from the workshops was the general sense of dissatisfaction with current working conditions, specifically regarding financial pressures and treatment timings. These findings are in line with previous studies [63]. Although direct access is not currently allowed for patients with statutory health insurance, there are pilot studies evaluating broader flexibility for physiotherapists in decision-making, and physiotherapists fulfilling certain criteria can apply for a release to directly treat patients who pay privately [64]. During the debate, such general professional-political discussions, which included academisation, were stated as possible facilitators for implementing change. From the participants’ perspective, they characterise a broader wish for change, which could also promote the adoption of new procedures like the STarT-Back-Approach.

### Strengths and weaknesses

We used a qualitative design which has previously been effective in exploring the perceptions of primary care physicians regarding the implementation of the STarT-Back-Approach [35]. Both researchers who coded the transcripts were present at all three focus groups, reducing the likelihood of misinterpretation since they both experienced the atmosphere and intonation during all the discussions. In general, it is recognised in qualitative research that facilitators’ experience and attitudes can influence the research process [65]. This cannot be measured but was considered by a description of the research team.

For all three focus groups, we reached good saturation in terms of the aims of the study to identify barriers to and enablers of the implementation of the STarT-Back-Approach [37]. The reliability for coding between the researchers was not evaluated, since it is understood that differences do not have to be considered as negative per se, but reflect perspectives of understanding which are always part of qualitative research [65, 66]. Indeed, analysis of the content of disagreement has been stated to be of greater importance than a calculated degree of concordance [67]. Therefore, categories with descriptions were developed and underpinned with quotes. Disagreements between coders were discussed in an intensive reiterative process resulting in a well-structured coding agenda.

In reflecting on our findings, it must be recognised that most German physiotherapists are trained at a vocational level, with fewer than 5 % trained with an academic qualification [68]. One limitation of this study might therefore be that the workshop participants on average were more highly qualified from an academic perspective than an average group of German physiotherapists. Still, participants trained at a vocational level clearly dominated in numbers, and most of the participants work in clinical practice.

### Research agenda

Like the findings from the primary care physicians, our physiotherapy participants feared that the use of the SBT might negatively influence their patient interactions [35]. Therapists’ perceptions of patients’ treatment expectations differed; whether this is true or not will be discussed with patients in a subsequent interview study, alongside other potential objections that patients may have about the UK’s STarT-Back-Approach. This information will be important for helping implementers to know how best to adapt these treatment approaches appropriately.

## Conclusion

Overall, our results identified a number of meaningful barriers to and enablers of implementing the STarT-Back-Approach. Enablers include the positive interest in and opportunities seen by the physiotherapy community in relation to this new treatment approach, the benefits expected from training in psychologically informed practice and the potential benefits for patients. However, realistic implementation of the intervention only seems likely if barriers around communication and education, especially for patients with complex problems, could be resolved, preferably by rolling out a post-graduate qualification programme. Moreover, external healthcare system constraints concerning the remuneration system and the flexibility of treatment time and frequency need to be overcome. Inter-professional collaboration, particularly with referring physicians, should be further improved by reduction of misconceptions about competencies, skills and attitudes towards each other’s profession.

## Additional files

Additional File 1: Interview Guideline. (PDF 75 kb)

Additional File 2: Subthemes. (PDF 190 kb)

## Abbreviations

CFIR: Consolidated Framework for Implementation Research
GP: General Practitioner
LBP: Low back pain
SBT: STarT-Back-Tool
STarT: Subgroups for Targeted Treatment

## References

[1] Hoy D, March L, Brooks P, Blyth F, Woolf A, Bain C (2014). The global burden of low back pain: estimates from the global burden of disease 2010 study. Ann Rheum Dis.

[2] Vos T, Barber RM, Bell B, Bertozzi-Villa A, Biryukov S, Bolliger I, et al. Global, regional, and national incidence, prevalence, and years lived with disability for 301 acute and chronic diseases and injuries in 188 countries, 1990-2013: a systematic analysis for the global burden of disease study 2013. Lancet. 386:743–800. 10.1016/S0140-6736(15)60692-4 PMC4561509 26063472

[3] Mafi JN, McCarthy EP, Davis RB, Landon BE (2013). Worsening trends in the management and treatment of back pain. JAMA Intern Med.

[4] Wenig CM, Schmidt CO, Kohlmann T, Schweikert B (2009). Costs of back pain in Germany. Eur J Pain.

[5] Swinkels IC, van den Ende CH, van den Bosch W, Dekker J, Wimmers RH (2005). Physiotherapy management of low back pain: does practice match the Dutch guidelines?. Aust J Physiother.

[6] Karstens S, Weiler SW, Frobose I, Peters-Klimm F (2013). Prescriptions in outpatient physiotherapy for low back pain - descriptive analysis to relate indication key and everyday impairment. Rehabilitation (Stuttg).

[7] Foster NE, Dziedzic KS, van der Windt DA, Fritz JM, Hay EM (2009). Research priorities for non-pharmacological therapies for common musculoskeletal problems: nationally and internationally agreed recommendations. BMC Musculoskelet Disord.

[8] Main CJ, George SZ (2011). Psychologically informed practice for management of low back pain: future directions in practice and research. Phys Ther.

[9] Karstens S, Hermann K, Frobose I, Weiler SW (2013). Predictors for half-year outcome of impairment in daily life for back pain patients referred for physiotherapy: a prospective observational study. PLoS One.

[10] Hill JC, Whitehurst DG, Lewis M, Bryan S, Dunn KM, Foster NE (2011). Comparison of stratified primary care management for low back pain with current best practice (STarT back): a randomised controlled trial. Lancet.

[11] O'Sullivan P (2012). It's time for change with the management of non-specific chronic low back pain. Br J Sports Med.

[12] Karran EL, McAuley JH, Traeger AC, Hillier SL, Grabherr L, Russek LN (2017). Can screening instruments accurately determine poor outcome risk in adults with recent onset low back pain? A systematic review and meta-analysis. BMC Med.

[13] Linton SJ, Nicholas M, MacDonald S (2011). Development of a short form of the Orebro musculoskeletal pain screening questionnaire. Spine (Phila Pa 1976).

[14] Hill JC, Dunn KM, Lewis M, Mullis R, Main CJ, Foster NE (2008). A primary care back pain screening tool: identifying patient subgroups for initial treatment. Arthritis Rheum.

[15] Kenny D, Ball J, Bloxham C, Cashmore G, Dick F, Kannan P (2015). An evaluation of the psychometric properties of the STarT Back Screening Tool - a systematic review. Physiotherapy.

[16] Institute of Primary Care and Health Sciences, Keele University, Keele/Stoke-on-Trent, United Kingdom. STarT Back: Translations. n.d. http://www.keele.ac.uk/sbst/startbacktool/translations/. Accessed 5 Feb 2017.

[17] Beaton D, Bombardier C, Guillemin F, Ferraz MB. Recommendations for the Cross-Cultural Adaptation of the DASH & QuickDASH Outcome Measures. Institute for Work & Health; 2007. http://dash.iwh.on.ca/sites/dash/files/downloads/cross_cultural_adaptation_2007.pdf. Accessed 17 Mar 2018.

[18] Aebischer B, Hill JC, Hilfiker R, Karstens S (2015). German translation and cross-cultural adaptation of the STarT back screening tool. PLoS One.

[19] Karstens S, Krug K, Hill JC, Stock C, Steinhaeuser J, Szecsenyi J (2015). Validation of the German version of the STarT-back tool (STarT-G): a cohort study with patients from primary care practices. BMC Musculoskelet Disord.

[20] Sowden G, Hill JC, Konstantinou K, Khanna M, Main CJ, Salmon P (2012). Targeted treatment in primary care for low back pain: the treatment system and clinical training programmes used in the IMPaCT back study (ISRCTN 55174281). Fam Pract.

[21] Sanders T, Foster NE, Bishop A, Ong BN (2013). Biopsychosocial care and the physiotherapy encounter: physiotherapists' accounts of back pain consultations. BMC Musculoskelet Disord.

[22] Foster NE, Mullis R, Hill JC, Lewis M, Whitehurst DG, Doyle C (2014). Effect of stratified care for low back pain in family practice (IMPaCT back): a prospective population-based sequential comparison. Ann Fam Med.

[23] Chenot JF, Scherer M, Becker A, Donner-Banzhoff N, Baum E, Leonhardt C (2008). Acceptance and perceived barriers of implementing a guideline for managing low back in general practice. Implement Sci.

[24] Werber A, Schiltenwolf M. Treatment of lower back pain-the gap between guideline-based treatment and medical care reality. Healthcare (Basel, Switzerland). 2016;4:44. 10.3390/healthcare4030044 PMC5041045 27417632

[25] van der Zee J, Kroneman MW (2007). Bismarck or Beveridge: a beauty contest between dinosaurs. BMC Health Serv Res.

[26] Freund T, Everett C, Griffiths P, Hudon C, Naccarella L, Laurant M. Skill mix, roles and remuneration in the primary care workforce: who are the healthcare professionals in the primary care teams across the world? Int J Nurs Stud. 2015;52:727–43. 10.1016/j.ijnurstu.2014.11.014 25577306

[27] Koch K, Miksch A, Schürmann C, Joos S, Sawicki PT (2011). The German health care system in international comparison: the primary care physicians’ perspective. Dtsch Arztebl International.

[28] Klemme B, Geuter G, Willimczik K. From an Academisation to the Profession. Physioscience. 2007;3:80–7.

[29] WCPT. United Kingdom: a profile of the profession. http://www.wcpt.org/node/25749/cds. Accessed 31 Oct 2017.

[30] WCPT. Germany: a profile of the profession. http://www.wcpt.org/node/25050/cds. Accessed 31 Oct 2017.

[31] Ärzteblatt. [Nearly nine millons privatly insured]. 2012. https://www.aerzteblatt.de/nachrichten/52395/Fast-neun-Millionen-Privatversicherte-in-Deutschland. Accessed 30.July 2017.

[32] Bury TJ, Stokes EK (2013). Direct access and patient/client self-referral to physiotherapy: a review of contemporary practice within the European Union. Physiotherapy.

[33] WCPT. World Confederation for Physical Therapy: Direct access and self-referral to physical therapy: findings from a global survey of WCPT member organisations. 2013. http://www.wcpt.org/sites/wcpt.org/files/files/Direct_access_SR_report_Jan2013.pdf. Accessed 26 Oct 2017.

[34] WIdO (2009). Therapy report 2009/2010.

[35] Karstens S, Joos S, Hill JC, Krug K, Szecsenyi J, Steinhauser J (2015). General practitioners views of implementing a stratified treatment approach for low back pain in Germany: a qualitative study. PLoS One.

[36] Kitzinger J (1995). Qualitative research. Introducing focus groups. BMJ.

[37] Onwuegbuzie AJ, Dickinson WB, Leech NL, Zoran AG (2009). A qualitative framework for collecting and analyzing data in focus group research. Int J Qual Methods.

[38] Karstens S, Steinhäuser J, Joos S (2013). Der STarT-Fragebogen. Ein Instrument zur abgestuften Therapiezuweisung bei Kreuzschmerzen pt_Zeitschrift für Physiotherapeuten.

[39] Sim J, Snell J (1996). Focus groups in physiotherapy evaluation and research. Physiotherapy.

[40] Dresing T, Pehl T. Practice book inview and. transcription. 2012; www.audiotranskription.de/praxisbuch Accessed 30 Oct 2017

[41] Mayring P. Qualitative Content Analysis. 2000. http://www.qualitative-research.net/index.php/fqs/article/view/1089/2385. Accessed 20 Dec 2014.

[42] Krippendorff K. Models of messages: three prototypes. In: Gerbner G, Holsti OR, Krippendorff K, Paisly GJ, Stone PJ, editors. The analysis of communication content. New York: Wiley; 1969.

[43] Damschroder LJ, Aron DC, Keith RE, Kirsh SR, Alexander JA, Lowery JC (2009). Fostering implementation of health services research findings into practice: a consolidated framework for advancing implementation science. Implement Sci.

[44] Sanders T, Ong BN, Sowden G, Foster N (2014). Implementing change in physiotherapy: professions, contexts and interventions. Journal of health organization and management.

[45] Wieringa S, Greenhalgh T (2015). 10 years of mindlines: a systematic review and commentary. Implement Sci.

[46] Francis JJ, O'Connor D, Curran J (2012). Theories of behaviour change synthesised into a set of theoretical groupings: introducing a thematic series on the theoretical domains framework. Implement Sci.

[47] R Development Core Team (2014). R: A C for statistical computing.

[48] Saunders B, Bartlam B, Foster NE, Hill JC, Cooper V, Protheroe J (2016). General Practitioners' and patients' perceptions towards stratified care: a theory informed investigation. BMC Fam Pract.

[49] Gurung T, Ellard DR, Mistry D, Patel S, Underwood M. Identifying potential moderators for response to treatment in low back pain: a systematic review. Physiotherapy. 2015;101:243–51. 10.1016/j.physio.2015.01.006 25769189

[50] Bath B, Grona SL (2015). Biopsychosocial predictors of short-term success among people with low back pain referred to a physiotherapy spinal triage service. J Pain Res.

[51] Balague F, Mannion AF, Pellise F, Cedraschi C (2012). Non-specific low back pain. Lancet.

[52] Synnott A, O'Keeffe M, Bunzli S, Dankaerts W, O'Sullivan P, O'Sullivan K (2015). Physiotherapists may stigmatise or feel unprepared to treat people with low back pain and psychosocial factors that influence recovery: a systematic review. J Physiother.

[53] Grafe M, Probst A (2012). Anforderungen an Physiotherapeuten im Handlungsfeld ambulante Physiotherapiepraxis. physioscience.

[54] Gardner T, Refshauge K, Smith L, McAuley J, Hubscher M, Goodall S (2017). Physiotherapists' beliefs and attitudes influence clinical practice in chronic low back pain: a systematic review of quantitative and qualitative studies. J Physiother..

[55] WCPT. What is physical therapy. 2016. http://www.wcpt.org/what-is-physical-therapy. Accessed 30 Oct 2017.

[56] Schoeb V, Burge E (2012). Perceptions of patients and physiotherapists on patient participation: a narrative synthesis of qualitative studies. Physiother Res Int.

[57] Roberts P (1994). Theoretical models of physiotherapy. Physiotherapy.

[58] Dixon M, Sweeney K. The human effect in medicine: theory, Research and Practice: Radcliffe Medical Press; 2000.

[59] Nielsen M, Keefe FJ, Bennell K, Jull GA (2014). Physical therapist-delivered cognitive-behavioral therapy: a qualitative study of physical therapists' perceptions and experiences. Phys Ther.

[60] Synnott A, O'Keeffe M, Bunzli S, Dankaerts W, O'Sullivan P, Robinson K (2016). Physiotherapists report improved understanding of and attitude toward the cognitive, psychological and social dimensions of chronic low back pain after cognitive functional therapy training: a qualitative study. J Physiother..

[61] Main CJ, Sowden G, Hill JC, Watson PJ, Hay EM (2012). Integrating physical and psychological approaches to treatment in low back pain: the development and content of the STarT back trial's ‘high-risk’ intervention (StarT back; ISRCTN 37113406). Physiotherapy.

[62] Scurlock-Evans L, Upton P, Upton D (2014). Evidence-based practice in physiotherapy: a systematic review of barriers, enablers and interventions. Physiotherapy.

[63] Barzel A, Ketels G, Schön G, van den Bussche H (2011). first Germany-wide survey of physiotherapists and occupational therapists on the professional situation part 2: Therapists’ Professional Life. physioscience.

[64] Wich M (2016). Räbiger J [Blank Prescription or Direct Access – Patients Must Be the Winners] physioscience.

[65] Meyer T, Karbach U, Holmberg C, Guthlin C, Patzelt C, Stamer M (2012). Qualitative research in health services research - discussion paper, part 1: what is the idea?. Gesundheitswesen.

[66] Breuer F, Mey G, Mruck K (2010). [Scientific theoretical principles of qualitative methods in psychology] Wissenschaftstheoretische Grundlagen qualitativer Methodik in der Psychologie. [Handbook qualitative research in psychology]: VS Verlag für Sozialwissenschaften.

[67] Barbour RS (2001). Checklists for improving rigour in qualitative research: a case of the tail wagging the dog? BMJ. Br Med J.

[68] Physio-Deutschland. Hochschul-Befragung 2013 des Deutschen Verbandes für Physiotherapie 2013. https://www.physio-deutschland.de/fileadmin/data/bund/Dateien_oeffentlich/Beruf_und_Bildung/Studium/PHYSIO-DEUTSCHLAND_Hochschulbefragung_2013_final.pdf. Accessed 14 Feb 2016.

