# Supplementary material for: Physiotherapists’ views of implementing a stratified treatment approach for patients with low back pain in Germany: a qualitative study
Source: BMC Health Serv Res. 2018 Mar 27;18:214. doi: 10.1186/s12913-018-2991-3 (PMC5872532; doi:10.1186/s12913-018-2991-3)
Supplement: Supplementary file 1 — Interview Guideline. (PDF 75 kb) [file 12913_2018_2991_MOESM1_ESM.pdf]

Physiotherapists’ Views of Implementing a Stratified Treatment Approach for Patients with Low Back Pain in Germany: A Qualitative Study

Karstens et al.

Additional file 1: Interview Guideline

Interview Guideline

Aim and basic information for participants

To explore participants' perspectives on implementing the STarT-Back-Approach and to understand the perceived potential organisational barriers to and enablers of using this approach within the participants' practices.

Discussions will be audio recorded.
The co-facilitator will take notes.
Data privacy: no names or personal information will be transcribed.

- Start recording -

Getting started: Allocation using STarT-Back-Tool

#1: What is your perception of the use of the STarT-Back-Tool in allocating patients with low back pain to the three different treatment approaches?

Main part: Implementation of STarT-Back-Approach in physiotherapy practice

#2.1: What requirements do you see for implementation of the high-risk approach?

#2.2: What requirements do you see for implementation of the low-risk approach?

Facilitators: qualification, training, structures, remuneration, collaboration, mentoring

#2.3: From your perspective: To what extent does the medium-risk approach differ from current physiotherapy practice in Germany?

Facilitator: Evidence-based practice

Ending: Round-up

Round-up by the facilitators, possible correction and reflection by participants.

Thank you for participating!

- End recording -
