# Supplementary material for: Physiotherapists’ views of implementing a stratified treatment approach for patients with low back pain in Germany: a qualitative study
Source: BMC Health Serv Res. 2018 Mar 27;18:214. doi: 10.1186/s12913-018-2991-3 (PMC5872532; doi:10.1186/s12913-018-2991-3)
Supplement: Supplementary file 2 — Subthemes. (PDF 190 kb) [file 12913_2018_2991_MOESM2_ESM.pdf]

Physiotherapists' Views of Implementing a Stratified Treatment Approach for Patients with Low Back Pain in Germany: A Qualitative Study

Karstens et al.

Additional file 2: Subthemes

Theme 100: Intervention Characteristics

#	Subtheme	CFIR	Paraphrase	Quote
101-1	Chances of implementation	I.IC_F	Re-thinking of therapists	PT1-3 I think it takes a lot of getting used to, but I don't see a problem from a therapeutic point of view, but it will take time to get used to. Because physiotherapy up to now has been quite intuitive, it's how physiotherapists work, not simply straight from study data.
101-2		I.IC_F	Daily business	PT2-2 I think [treatment as described for the STarT-Approach] that´s what we´re doing day after day anyway.
102-1	Matching patients	I.IC_C	Systematic optimised therapy	PT7-1 I think it's a good approach and a necessary approach. I think that the patients will benefit from it too, to provide them with an optimised therapy.
102-2		I.IC_C	New matched approach	PT3-2 I think what's really fundamental is the differentiation in treatment. That chronic patients get a different treatment over time but also covering different contents. That's an important approach that currently probably doesn't happen in many practices or treatment centres.
103-1	Benefits of classification	I.IC_C	Strength of objective subgroups	PT3-1 I think it's good, and I like it that these psychosocial factors are given some relevance, because I think that it can be critical for the patients. The pain can be really acute, but one patient copes with it quite easily, but then there are others for whom quite a low level of pain has really drastic effects, for example lower back pain, and that's an important point.
103-2		I.IC_C	Increasing effectiveness	PT8-1 In total I endorse the STarT-Back Tool, because low back pain, quite a big topic … You can sub divide it in low back pain which is not that severe and in low back pain which is really bad. And therefore, being effective and also to reduce costs I think it´s quite good to subgroup. That you can treat effectively- let me put it that way.

#	Subtheme	CFIR	Paraphrase	Quote
104-1	Implementation of classification	I.IC_D	Gut feeling vs classification	PT 1-3 I find it hard to pigeonhole patients like that. […] but I don’t see a problem from a therapeutic point of view, but it will take time to get used to, because physiotherapy up to now has been quite intuitive.
104-2		I.IC_F	Application of subgrouping	PT2-3 Because I do have a pigeonhole problem with patients, too. And I think that I peg patients way to early. But since I call it differently, it´s not associated that negatively anymore. Because subgrouping sounds much better (laughter). But it´s not that absolute ... has been demonstrated, not only in low back pain but also with other complaints, that´s important to categorise patients, yes.
105-1	Structure STarT-Back Tool	I.IC_G	Length of the tool	PT4-1 Well, I think it’s good and I think it’s good that it’s manageable and not too long. Even though there are only nine questions, it still manages to address many of the issues, for example I can find out how active the patient is, how much pain they are in, and even how much their everyday life is restricted. I think it’s good.
105-2		I.IC_G	Cover of sufficient details	PT1-1 I think the way it’s basically organised is good too, […] I don’t know whether perhaps you would need to identify what sort of patient you’re dealing with based on the pain symptoms. Just classifying without necessarily taking state of mind into account I think is okay. But whether it’s enough, or whether perhaps there ought to be more questions, or perhaps more precise questions about pain maybe, I don’t know.
106-1	Consumption of time	I.IC_F	High practicability	PT2-3 Well, I think it is really positive is that there are only nine questions. Because there are other questionnaires that are significantly longer and therefore more difficult to evaluate. At first glance, and without having worked with it yet, this looks simple and practical. The fact that you get straight on the right track.
106-2		I.IC_F	Shortening therapy	PT1 -3 Maybe it reduces the treatment duration, as it clarifies in advance and covers really a lot. Possibilities [obstacles] what else could go on top.

#	Subtheme	CFIR	Paraphrase	Quote
107-1	Advantages of STarT-Back Tool	I.IC_C	Facilitation of treatment	PT3-3 What's really interesting about a questionnaire like this is that it means you've got a totally different starting point with the patient, when you give him or her something like this. And of course the patient gets a completely different focus, no more 'I'm going to get pulled around the whole time', to put it negatively, but instead a self-orientated approach looking after yourself, something we need more of nowadays. The fact that the patient is proactive and we can get away from never-ending treatment sessions, yes, I can really see how a questionnaire like this could work well.
107-2		I.IC_C	Covering undetected aspects	PT1-1 […] because with some patients things emerge after the second or third treatment which were never on the table to begin with, maybe because you forgot to ask certain questions when you were doing the case history. But will this questionnaire take over, this is what I wonder, will it take over […] or is that going a bit too far?
107-3		I.IC_C	Self-reflection of the patient	PT2-1 When I read this I got the impression, that the patient can reflect himself briefly, why he got here, or how he is dealing with his pain, and the importance of the pain for him. Because the questions are very specific and otherwise wouldn't be asked a lot and especially not from the medical doctor, that´s quite often my impression. But thus, the patient is just a bit more aware, also due to the questionnaire, of the reason attending the clinic.
108-1	Intensity assessment and advice	I.IC_F	Functional assessment	PT7-2 And I don't know if I as a [...] physiotherapist would act like that. I would at least say: 'I see, you have leg pain, can you stand on that leg?' Well, I would like to get a bit more assurance. That's what I always do, getting a functional impression.

#	Subtheme	CFIR	Paraphrase	Quote
108-2		I.IC_F	In depth assessment	PT8-1 I don´t know if I would be able to only do the 'assessment and education' thing, to tell them: 'now you have to join a sports club, do something, have a look at the exercises [they offer] there.' That´s not sufficient for me. I need the entire thing, you have to assess and detect the reason for it, maybe, do this and this, but just saying this is the information you can get it out of the book and good bye.<br>PT 6-1 At least some posture corrections, the basics you do as a physio. PT8-1 Yes, this is lacking.<br>PT6-1 I´m missing that, too. If you only see him once for 30 minutes.
109-1	Objectiveness	I.IC_C	Unbiased assessment	PT1-3 Somehow you just get this from someone. You see a patient and then you reckon you know what’s wrong. You just have to hold yourself back for a moment, if you’re any good, then you think things over, leave it all open, and maybe it helps you, even if you get some real surprises when a patient ticks completely different answers to the ones you expected.
109-2		I.IC_C	Increasing objectiveness	PT2-3 As for the psycho-social side of things, you get a picture of what a patient is like when he comes in. And the more you work with them, the more you think you’ve got the picture of them, but of course that’s all very subjective. This questionnaire helps people to be more objective about how they classify a patient. I mean I still try to think when I see a patient ‘have they got more of a physiological or more of a psychological problem?’, but some come in flagging something obvious or something. But to get away from that, I’d like to get away from the intuitive approach, to be more objective.
110-1	False classification	I.IC_G	Certain type of patients	PT4-2 But I think every one of us knows this sort of nice patient who can still function but still suffers from something. If I look at these aspects: 'I worry often' and 'it´s terrible and it´s never going any better', these patients, I could imagine, could head into the wrong direction ... considering a certain type of person.

#	Subtheme	CFIR	Paraphrase	Quote
110-2		I.IC_G	Trust in answers	PT7-2 I think that a patient says 'I worry' or 'I do worry often' only if it's really severe. [Then] you know, that the patient feels like this, most patients have a feeling for themselves. But if they really tick yes, that´s the criterion, indicating that they are really low-risk or even in medium risk.
111-1	Timepoint of STarT-Back Tool application	I.IC_D	Prior to PT setting	PT4-2 Ideally in future the patient should bring the completed questionnaire with them. I would like it like that. […]<br>PT4-2 Yes, because then, when he comes along the first time and I see him, then I haven't got time to explain everything to him again.
111-2		I.IC_D	Waiting area	PT3-2 The best way to organise things might be to give the patient this questionnaire in the waiting area beforehand so the he or she can fill it in for themselves, then they come on in with it already completed. You could even put it to one side to start with, do the normal case history as you usually do. Then look at the questionnaire to see what comes out and then plan the way forward accordingly. If you did that, then I think you wouldn't be in this mess with asking each of the questions one after the other.
111-3		I.IC_D	Interactive approach	PT7-2 I ask, because of the questionnaire, is that asked personally in England, does it make a difference if someone is like reading it out quote unquote and asks?
112-1	Treatment description	I.IC_G	Flexibility	PT2-3 But there's enough scope I think [for individual decisions] within the three branches.
112-2		I.IC_G	Systematic procedure	PT6-1 it´s positive, in my opinion, too, that there is a systematic approach, having a kind of guideline. A starting point.
113-1		I.IC_G	More specific details required	PT1-1 […] the thing I miss a bit is the particularisations. There is an evidence based course of physiotherapy but I'm not quite sure about whether particular exercises or manual treatment techniques are to be used on patients.

#	Subtheme	CFIR	Paraphrase	Quote
113-2	Standardisation	I.IC_G	Manual therapy	PT2-3 […] what is manual therapy. Has that been set down or is it still to be set down? Or is it just the framework for individual intuition. There are people who define it as just soft tissue techniques or manual massage. We've read just now that massage shouldn't be done. And that's why something like this is important. How to I standardise, without narrowing them down too much, the various things.
113-3		I.IC_G	Neurological examination	PT4-2 One thing I often ask myself is: is it planned to do a neurological investigation as standard, or are there criteria and if so which? Because why should I do a neurological investigation on a patient who has back pain. There is no indication given there to do. Differential diagnosis for a hip problem you have to ask yourself: 'do I have to rule out a problem with the hip for every patient and if I do, how?' Any suggestions?
114-1	Treatment duration	I.IC_B	Less treatment required	PT1-1 I don't understand why in the study they come up with the figure of six treatment sessions, because that's not many for us. We have twelve or eighteen or more. Let's assume something happens. Why was a measurable improvement in the level of pain achieved. But perhaps that can be traced back to the way the questions were put in the first place because with some patients it's really the case that things emerge after the second or third treatment session that simply weren't in the mix at the start.
114-2		I.IC_B	Astonishment treatment amount	PT3-3 [...] if I would have such an improvement after four treatment sessions, not needing 18 and having no difference [in treatment outcome].

Theme 200: Setting

#	Subtheme	CFIR	Paraphrase	Quote
201-1	Chances of implementation	III.IS_D	Re-thinking	PT2-1 I think that´s a thrilling topic, and also necessary, but from the view of our own professional identity we really do something completely different.
201-2		III.IS_A	Already similar clientele	PT7-1 I don´t think that´s something totally different from what we are currently doing, because we have patients with either low or high risk, or who already had become chronic.
202-1	Remuneration	II.OS_D	Comparable tasks	PT6-1 If we're really honest about this, we're moving much more in the direction of becoming a sort of health coach. A Health Coach expects different things to what a physiotherapist does. [...] but it was 40 Euro an hour. It's meant to be 45 minutes, so if I imagine I've got a practice, then for the business side of things a Health Coach costs a hell of a lot of money, so how would that work for the practice and what would you get out of it.
202-2		II.OS_D	Responsibility related remuneration	PT1-1 Perhaps the pay would be better then ... Because then you would have a lot more responsibility. PT6-1 yes, getting 14 euro and 10 cents you can't provide it. Especially not forty-five minutes.
202-3		II.OS_D	Overall frustration	PT1-3 I think reimbursement is rather cause of frustration for all of us therapists [laughter], we are convinced that we don't get paid to an extent we think we are qualified [...]. With these tasks [described for the STarT-Approach], with these additional qualifications, physiotherapy is gaining more, dramatically more importance and thus deserves a higher reimbursement.
203-1	Profitability outer setting	II.OS_D	Lump-sum payment	PT7-2 […] Just that it doesn't matter if you talk to him [the patient] or if you put him on a bicycle, in terms of the concept, it shouldn't matter. That has been my request for a while, however.

#	Subtheme	CFIR	Paraphrase	Quote
203-2		II.OS_D	Business orientation	PT7-2 I think this is interesting, whether it's possible to do this in practices that are business orientated and want particular margins, whether it's feasible.
204-1		III.IS_D1	Impracticability of current system	PT4-3 you have to cheat to treat patients accurately.
204-2	Profitability inner setting	III.IS_C	Patient trust	PT2-2 I think word gets around that it's not just about getting as many referrals as possible and treating the same thing every week for years [that is positive]. Instead you say 'this is what it's about, now off you go to the sports club' or something like that.
204-3		III.IS_F	More patients	PT2-3 Well, I wouldn't be afraid, as we've discussed before, that this model leads to seeing less patients. Because I think, if this gets established, I see more patients at the end. Thus if I can't see every single one that often, but I'm not afraid to see less patients. But I think, that a lot of PTs, even if they are self-employment or being afraid of, because such a long-term patient is a fixed [source of income].
205-1	Current System	III.IS_D1	Frustration with current system	PT3-3 I don't know whether it's because I'm more aware of it, but I do find that patients where psycho-social factors have an influence are clearly on the increase. Or perhaps I'm just more aware of them, and according to how important or critical these factors are, it's frustrating to treat them sometimes, because you don't have or choose the right approach. It could be patients coming on a repeat prescription to be treated and that can become stressful for both parties in the long run. That's why I think it's a good idea to think through the whole concept that we have in Germany, and re-examine it. [...] How it's carried out, the restrictions for doctors and suchlike.

#	Subtheme	CFIR	Paraphrase	Quote
205-2		III.IS_D1	Management demands	PT6-2 Yeah, I think so too, what you just said is really a dream, that you have a free choice; what really bothers me about the [referral process] are these constant interruptions. The other way would work better for the health insurances, wouldn't it? They would also save money, if you wouldn't rush through things in a flash, but say: 'let's make intervals [between treatment sessions] longer.
205-3		III.IS_D1	Downsize of physiotherapy	PT3-3 […] the more you, as a physiotherapist, address these psycho-social aspects, don't you risk rationalising yourself out of the picture?
206-1	Professional policy outer setting	II.OS_D	Framework requirements	PT2-2 [...] the professional framework requirements have to be set, to be able to use the questionnaire and this stratification. Well, therefore to say, now he is part of that risk group and I have the freedom to say, that he gets just one session and of course you need training for that, that you are able to implement it.
206-2		II.OS_A	Interest of insurance companies	PT3-1 I think the health insurance companies could be really interested in this being the first thing to give to the physiotherapists. Because it's all about how conditions becoming chronic and they're the people who have to pay for everything afterwards. The quicker they're in a position to say: 'OK, this case can be dealt with quickly but this other one needs more time', then the lower the costs they incur at the end of the day.
207-1	Manual therapy	III.IS_C	Unprecise definition of PT content	PT2-2 No, but it's actually quite difficult when some asks me what is manual therapy. I am hard pushed to explain sometimes because I say: what's the difference and can you separate it in your mind – you can't can you? If someone is doing physiotherapy, and that's in your mind, you can't just say well today I'll just do that, the other thing hasn't been prescribed so we won't do it.
207-2		III.IS_A	Heterogeneity of manual therapy	PT3-3 We do have manual therapy, and you can hardly compare the different styles [concepts], well, Kaltenborn, Maitland, […].
208-1	Interprofessional collaboration	II.OS_B	Interprofessional collaboration	PT3-1 I don't know how it's done in England, whether each doctor sends a patient to this practice or a different one on a whim, or whether there are interdisciplinary

#	Subtheme	CFIR	Paraphrase	Quote
				tie-ups with a particular physio who is at the same level that were talking about in this seminar. And then say: 'OK, I'll send this patient there and I'll get a call back at some stage and it's not a story that's been moulded to fit the prescription,' but you can honestly say 'I've classified the patient like this, should we proceed like this?' And he [physician] gives it the nod because he knows what I'll be doing next, like that. I don't think you should scatter this about so that everybody gets involved; better to work with an established partner.
208-2		II.OS_B	Development of health care centres	PT2-2 Perhaps we need to develop centres where you say 'OK there's a place there where patients with back pain can go' and you work according to this concept and a whole lot of things are on offer. I could see that would work.
209-1	Collaboration with GP	II.OS_B	Facilitating GP relationship	PT2-3 Well, I could see that the questionnaire could bridge the collaboration gap to the doctor. That a patient is actually referred to us at an earlier stage, that the Physio is rather seen as a specialist. I think that's a good opportunity - exactly.
209-2		II.OS_B	Shared decision making	PT4-2 I think one prerequisite has to be that the doctor is on the same page and is thinking along the same lines […] I could imagine setting aside blocks of 2 or 3 hours doing one of these after another and then deciding in consultation with the doctor what we're going to do. The question remains if I as a therapist have to decide that on my own, or wouldn't it be better to decide that as a team, eventually even with psychologists from the beginning. And, in my opinion, I would strongly prefer to do it rather together in a team as doing it on my own with sole responsibility. Because the patients often just don't need only us.
210-1	Implementation of interprofessional collaboration	II.OS_C	Importance	PT3-2 I think the interdisciplinary thing will become more and more important. It's just difficult putting it into practice in the current set-up.
210-2		II.OS_C	PT referral conflict	PT6-1 It's just, the question remains if the doctor is interested in it, if you think of all the individual self-pay offerings [in physician clinics] and if you ask the patients what they had already tried before they end up with us. Then they did whatever

#	Subtheme	CFIR	Paraphrase	Quote
				kind of therapies for a lot of self-paid money, before a doctor has the idea of referring to physiotherapy, which is paid by the insurance company.
211-1	First contact	II.OS_C	Autonomy	PT1-2 But, I think that, would it be just **the** opportunity, that not the doctor has to decide what the physiotherapist has to do.
211-2		II.OS_D	Referral after first contact	PT7-1 But just looking at this again whether this is do-able with first contact for the physio, which I would really welcome. I can imagine this system of referral both ways. Whether I refer the patient after assessing the info in the questionnaire or whether the doctor does it and refers the patient to me it doesn't really matter<br>PT4-1 All the same it would be good for both to know him, it's an advantage, then giving feedback is simpler, if I say 'I've done this or that', or whether the doctor says 'I've done this or that', when the patient comes along. For this reason it's best if there is an interdisciplinary cooperation.
212-1	Role of PT	III.IS_D4	Involved in diagnosis	PT3-2 I think the difference is that with a questionnaire or screening like this we the physiotherapists move into the diagnostic process instead of being involved only in the therapeutic process. And because of this we have a greater responsibility to the patient.
212-2		III.IS_D4	Responsibility	PT21-2 So put more responsibility in the hands of the physiotherapist instead of just 'here's the referral: six times 20 minutes of this'. If I'm lucky, the doctor's diagnosis will be on there, and it sets out what I have to do, but with this I have the chance when the patient comes to me to assess for myself, I've got a number of treatment approaches to choose from, and make my assessment of the patient based on evidence that I now have, so if I have the chance to make more decisions for myself, then I'm all for it.
212-3		III.IS_D4	Freedom of treatment scope	PT1-1 If it is managed by the physiotherapist, because I really see these patients who don't need six sessions every so often. They only need one or two sessions, or most times, it is already fine when they come and see me. Yes, then it would

#	Subtheme	CFIR	Paraphrase	Quote
				be ok, if I can decide it or, I don't mind, after consulting the doctor. But then really have the freedom of decision making, that would be ingenious.
213-1	Competences perceived by others	II.OS_C	PT decision making	PT6-1 But then along come the doctors, I can imagine, and say: 'can a physiotherapist make a decision on that?' Or is it enough again to assess by using the questionnaire […].
213-2		II.OS_C	Lack of trust of physicians	PT7-1 […] and our everyday reality is that there is little confidence in our capabilities […] and in my opinion, this approach enhances our own confidence as well as the physicians trust in us, that the patients receive appropriate physiotherapy treatment.
214-1	Role of PT within the healthcare system	II.OS_C	Advertisement with STarT	PT1-3 If the questionnaire really is so good, works so good, that would indeed be a first flagship. Meaning, that in medicine or even politics they would say: 'look how important physiotherapy is!' By taking on additional tasks and getting extra qualifications physiotherapy takes on a much more important role, becomes much more important and so earns a right to be better remunerated.
214-2		II.OS_D	Anticipated appreciation by patients	PT3-1 [...] but if the patient's ideas and expectations belong to evidence-based medicine then the patient should be a part of the decision, whether the physiotherapist is not a part of the process, or if that's even important to him. I would expect that for most patients the physiotherapist does play a part.
215-1	Patients' self-commitment	II.OS_A	Intrinsic motivation	PT5-1 I mean it depends on the patient. If he is really motivated and wants to recover quickly, then things will go faster […] but you have to call it what it is, for those people who enjoy being given a sick note and spending a week or two at home [...]. You have to call it what it is, there are people who like it.
215-2		II.OS_A	Passive patient expectations	PT7-2 Because it's like that, some patients do have different expectations: 'she has to fix it!' Taking into account that someone with a high risk could therefore have a different expectation.

#	Subtheme	CFIR	Paraphrase	Quote
216-1	Patients' views on treatment scope	II.OS_A	Claim for total amount	PT2-1 And of course the way referrals are given will have to be different. If you've got these six treatment sessions for say 15-20 minutes, and you have a patient who arrives with the idea that he has a right to get three referrals – three times six treatments then we'll be stuck in the middle thinking 'actually you don't need 18 treatment sessions'.
217-1	Patients' rethinking	II.OS_A	Astonishment	PT7-2 Because I'm wondering how I find that and I have patients, who would be really astonished, if I would just ask: 'show me how you elicit the pain or when it's the most severe?'
217-2		II.OS_A	Readjustment	PT2-2-There needs to be a change of attitude, I think, not only with the doctors, but also those who come to see them. I think it's a totally different approach and no one is used to it so far. It could be met with amazement to start with." PT7-2 [roleplaying] 'She didn't even let me get undressed [laughs]. She just asked me things.'
218-1	Patients' expectation on passive treatment	II.OS_A	Expectation massage	PT1-1 But perhaps it's what patients expect, I mean lots of patients come along expecting massage, don't they? And if I just give information and what I've found [during assessment] then perhaps the patients are going to think: 'you haven't really helped me here at all.'
218-2		II.OS_A	Already active patients	PT2-2 Or by all means some people who say: 'yes, yes I do really a lot in the gym, however, but please give me a massage.' And you just think: 'Why - and now?'
219-1	Patients' reaction to classification	II.OS_C	Loss of patient to other health care practitioners	PT2-2 But we have to look at this, when we've put them in the wrong category and they go off and look for something [other treatment] else.
219-2		II.OS_A	Against patient expectations	PT6-1 If I was a patient, perhaps I would expect or want at least that the physiotherapist immediately shows me something. Even though I consider that not as bad, the patient might see it totally different. He might have a total different expectation of his complaints compared to the outcome of the questionnaire.

#	Subtheme	CFIR	Paraphrase	Quote
220-1	Misinformation	II.OS_A	GP influences expectations	PT2-2 [...] the GP told them [the patients]: 'yes, then the therapist will pull you apart.' So! That makes me thinking they kind of fuel their expectations, that make you think, well do I think that makes sense? What next? Then you have to start all over again with education and advice.
220-2		II.OS_A	Biomechanical diagnosis	PT7-2 True, sometimes they say that they have a locked SIJ and it's obviously written that you have to correct it. If that's what they really need or not that's another story. Maybe it's not locked though, but that's simply the … Well the patients were lead with certain expectations about the following [treatment] content.
220-3		II.OS_A	Internet	PT2-2 Perhaps, maybe with some [patients], doctors fuel the expectations or they [the patients] get misinformation on the internet [...].
221-1	Patients' status	II.OS_A	Ending therapy	PT2-2 Basically we are getting to a certain point when we have to realise that [the patients] shouldn´t see a physio anymore, rather sending them to a back pain prevention programme or a back exercise group.
221-2		II.OS_A	Acute versus chronic first	PT4-2 The acute case has probably got a chance if you read only the literature that says they get better on their own [laughter amongst the group] [..] and the other one runs the risk of getting worse if he has to wait 19 days for an appointment. You have to teach your team to take the chronic case first, which of course doesn't happen in everyday practice because the other one is in pain.
222-1	Requirements for staff	III.IS_A	Problem due to low qualification level	PT4-2 We've already used this questionnaire in our Practice. We did it three years ago, […] and a massive problem was that colleagues were at different levels as regards how to deal with the information that came out of it and so we put it aside because the colleagues who were now working in the area of chronification didn't have the means to get any benefit out of it for their dealings with patients.

#	Subtheme	CFIR	Paraphrase	Quote
222-2		III.IS_A	Congruent team	PT7-2 It's only a feeling, but I think it would be easier if this was part of an agreed procedure for the whole Practice. That would mean that all the patients would be processed like this. If you had colleagues who weren't assessing patients like this, then they would, how can I say it, come under a sort of peer group pressure.
222-3		III.IS_A	In-house exchange	PT3-3 I think that in England you've got this thing with Juniors and Seniors so that in effect you have more opportunities as a beginner in your profession to exchange with older colleagues and such and I see a potential for that in these groups. As a Junior you can look after the low risk cases and then later on becoming a Senior you can work with the high risk cases. Perhaps a structure like that would be desirable.
223-1	Organisation in clinic	III.IS_E2	Media room	PT2-3 More pragmatically considered, that shouldn't be a problem anymore. That if you open a laptop and play a video, well. But from an economic point of view, it's harder to find a place. I can't treat other patients at the same time. Or should he take the laptop in the bathroom or what else? In the waiting area he might disturb others, or isn't focussed on it. But that's it, we are back at the remuneration yes. Do you have a separate room, are you sitting next to him, do I have to sit next to him, if he doesn't understand parts of it or something like that. Is he allowed to ask questions afterwards?
223-2		III.IS_E2	Privacy	PT3-3 Who has got these sort of facilities [needed for the STarT-Approach]? Who's got a room with a door? And not a room with three benches and a curtain [in between], for example.
223-3		III.IS_E2	Individual rooms	PT2-2 If you go down this route, then it could be that a physiotherapist will need his own room. And has the right equipment available there. Then it won't happen, that all the rooms are occupied. You could manage that every therapist has his room.
224-1	Allocation of competencies	III.IS_D2	Within the clinic	PT1-3 Even not every physio has to be a specialist of some sort, I think that's clear. Perhaps it would be sufficient when in your centre there would be one or

#	Subtheme	CFIR	Paraphrase	Quote
				two experts who do the first session and then, depending on the risk status, pass the patient on a junior therapist according to the therapeutic approach, recurrence rate.
224-2		II.OS_C	Covering all treatment pathways	PT3-3 Well. But one day, when it's established, do I give the questionnaire to the patient and then fill it together with him. Do I have to be able to do all of it? Or do I refer him to the next clinic? Over there, there are the high risks ones, that's not feasible, though.

Theme 300: Characteristics of Individuals

#	Subtheme	CFIR	Paraphrase	Quote
301-1	Role of physiotherapist	IV.CoI_B	Coordinator	PT3-3 So I could see myself in the role of somebody pointing the way forward, a sort of coordinator like care-planning in the hospital is done.
301-2		IV.CoI_B	Referring	PT1-1 Yes, if a physiotherapist would guide this [treatment allocation], maybe this wouldn't be too bad.
302-1	Competencies in management of complex patients	IV.CoI_B	Preference interdisciplinary team	PT5-1 I think that with low or medium risk it's perfectly possible to manage on your own. But if you have high risk patients, I think I would prefer to be part of an interdisciplinary team which I could swap ideas with.
302-2		IV.CoI_E	Confidence dealing with psychosocial aspects	PT6-2 [...] and I think we are actually already trained. To take into account this aspect with the high risk this psychosocial aspect. I see this again and again that it's more useful to spend 20 minutes talking and not doing manual therapy.
303-1	Work experience	IV.CoI_E	Treatment routine	PT1-3 I see things differently, in that they [physiotherapists] get into a rut over the years, and they treat not according to what they find but to standard cases and believe that they know what the patient has.
303-2		IV.CoI_E	Post-training qualification	PT4-3 Yes, and learning it over the years. You are not a good therapist after finishing training, actually experience only comes over the years.
304-1	Addressing psychosocial aspects	IV.CoI_E	Need for work experience	PT4-3 I think, yes, then it is exactly right to work on psychosocial aspects, too. That's what you only learn working with the patient, you only learn by experience.
304-2		IV.CoI_E	Difficulties learning communication strategies	PT3-2 But it's definitely true that it's something that not everybody can do [skilled communication] and it's hard to learn it on a theoretical basis. I think you'll need supervision on top, [...].

#	Subtheme	CFIR	Paraphrase	Quote
305-1	Commitment	IV.CoI_E	Need for idealism	PT1-3 Well the Physiotherapist in Germany who brings a certain idealism to the table has developed the basic qualification necessary. Because he doesn't get enough of that from school. The person [physiotherapist] who is interested will have taken the trouble to get educated to a higher level and got better qualified. And made sure he is better informed which in turn means taking on a lot of expense on his own.
305-2		IV.CoI_D	Commitment	PT1-3 I think that's right as well. Too few physiotherapists who are reasonably committed. When I say committed, I mean the ones who regularly upgrade their skills and aim at offering better therapy. Or who aren't trying to make themselves look good.
306-1	Academic qualification	IV.CoI_E	Not part of past vocational school training	PT2-2 Okay, during my academic training we addressed this topic. But before, it was not such a big deal: communication, education, … but when I look back to my vocational training there was none of it.
306-2		IV.CoI_E	Benefit of academic education	PT4-3 When we are talking about vocational training. And I've just visited two, it's all technique, technique, technique. Now there are academic programmes in addition, 40 or 50 of them [at universities], which have more of that stuff included: clinical reasoning and all this stuff. There are vocational schools which still don't teach evidence-based working. Yes, I guess in this respect an academic graduate has an advantage.
307-1	Self-efficacy	IV.CoI_B	Above average skills	PT1-3 But this, yes the tests, restricting its implementation more and more. There are really high requirements to meet to really use the questionnaire as well as realising the concept as a therapist. Well, the run-of-the-mill physiotherapist might be out of his depth, mightn't he? Even on a medium level risk, [...] the average physio can't do differential diagnosis, can he?
307-2		IV.CoI_B	Qualification thru training sufficient	PT7-1 I think it's not so different from what we currently do, [...] and to answer the question under what circumstances I could imagine it: certainly a training like that [described for the STarT-Approach] would be appropriate.

#	Subtheme	CFIR	Paraphrase	Quote
308-1	Acceptance by novices	IV.CoI_E	Assisting detection yellow-flags	PT4-2 [...] - being an experienced therapist - I shouldn't need to use the questionnaire to screen for the yellow flags. Whereas perhaps someone new to the job might prefer to do this using the questionnaire, because he can spot it better that way at the end of the day.
308-2		IV.CoI_E	Guiding novices	PT2-2 I think it is a good way of doing it. Thinking about myself being a novice, it really helps me if I could give it to the patient and see to which group he fits. Do I have to pay more attention to that, or is it someone with very little, what means little pain but, who is a low risk one, then as a novice I think it's really convenient and in general speaking, I like the concept.
309-1	Training of psychosocial aspects	IV.CoI_E	Post-graduation training preference	PT2-2 To address the patients' needs, empathy and …, guiding them without imposing your own goals. That you really try to develop the goal collaboratively with the patient. And I think in this respect communication is a huge topic for physios. Because, usually you do manual therapy, neurological post-graduate training and there is little approval, simply put you don't get continuing education credit-points [by health insurances].
309-2		IV.CoI_B	Necessity for training courses	PT3-3 The basic assessment [described for the STarT-Approach], well, reflecting my vocational training, I wouldn't have seen myself qualified to do this. That only came later. I don't know what it's like nowadays. I hope it improved, specifically neurological examination, to differentiate and so forth.
